# Assessing the Wnt-reactivity of cytonemes of mouse embryonic stem cells using a bioengineering approach

**DOI:** 10.1016/j.xpro.2021.100813

**Published:** 2021-09-15

**Authors:** Sergi Junyent, Joshua Reeves, Shukry J. Habib

**Affiliations:** 1 Centre for Stem Cells and Regenerative Medicine, King’s College London, London SE1 9RT, UK

**Keywords:** Cell Biology, Cell culture, Developmental biology, Microscopy, Signal Transduction, Stem Cells, Biotechnology and bioengineering

## Abstract

These protocols investigate the interaction of cytonemes with localized Wnt. Cell-niche signaling between naive or primed mouse embryonic stem cells (ESCs) and either Wnt-secreting trophoblast stem cells (TSCs) or Wnt signals tethered to microbeads can be scrutinized *in vitro*. This approach analyzes cytoneme reactivity during Wnt-interaction initiation, Ca^2+^ transients at Wnt-contacting cytonemes, and subsequent pairing between ESCs and Wnt-sources. This pairing interaction is crucial to synthetic embryogenesis; hence this protocol is effective for *in vitro* studies of developmental biology.

For complete details on the use and execution of this protocol, please refer to Junyent et al. (2020, 2021a, 2021b).

## Highlights

• These protocols investigate the interaction between the cytonemes of ESCs and Wnt sources
• The reactivity of ESC cytonemes to TSCs or immobilized Wnt sources is measured
• Localized Wnt-mediated Ca^2+^ transient generation in ESC cytonemes can also be quantified
• Both methods can be used to study cytoneme function differences in naive and primed ESCs

## Graphical abstract

**Figure:**
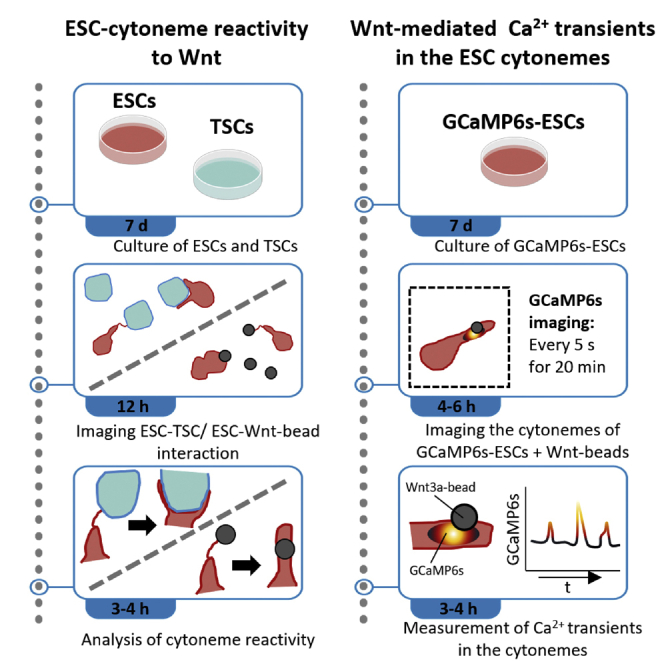

## Teaser

These protocols investigate the interaction of cytonemes with localized Wnt. Cell-niche signaling between naïve or primed mouse embryonic stem cells (ESCs) and either Wnt-secreting trophoblast stem cells (TSCs) or Wnt signals reconstituted on microbeads can be scrutinized *in vitro*. This approach analyzes cytoneme reactivity during Wnt-interaction initiation, Ca^2+^ transients at Wnt-contacting cytonemes, and subsequent pairing between ESCs and Wnt-sources. This pairing interaction is crucial to synthetic embryogenesis, hence this protocol is effective for *in vitro* studies of developmental biology.

## Before you begin

The protocols below describe the specific steps required to prepare the cells used in our experiments, including ESCs and TSCs, as well as the steps followed to transition ESCs to primed ESCs, and how to prepare ESC lines expressing GCaMP6s or Ftractin-mRuby3. Also, we briefly describe the preparation and testing of immobilized Wnt3a microbeads, although a comprehensive step-by-step protocol can be found in (Lowndes et al., 2017)

### Culture of mouse embryonic stem cells (ESCs)

**Timing: 5 days to several weeks**

This protocol describes the thawing, culture and passaging of ESCs. The use of low-passage ESCs (below 30 passages) is recommended. ESC colonies should be routinely checked for karyotype and the expression of pluripotency markers (e.g., high Alkaline Phosphatase staining, high Nanog and Klf4 expression and low Otx2 and Fgf5 expression) (Junyent et al., 2020; Martello and Smith, 2014; Ying et al., 2008).

**Figure 1:**
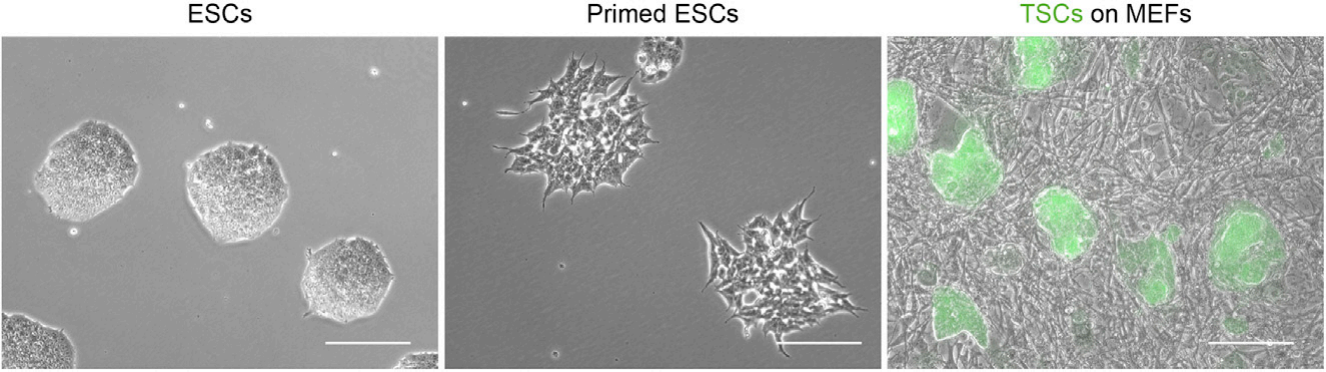
Representative images of ESC, primed ESC and TSC colonies TSCs express GFP (green) and are cultured in a layer of mitotically inactivated MEFs. Scale bars, 100 μm.

1. Thaw a vial of cryopreserved ESCs:
  a. Transfer the vial from a long-term storage unit (e.g., liquid nitrogen (LN2) tank) to a polystyrene box containing dry-ice pellets.
  b. In a tissue culture hood, prepare a 15 mL Falcon tube with 10 mL ESC basal media (see materials & equipment for composition).
    **CRITICAL:** ESCs are very sensitive to DMSO present in the freezing media, which can affect their viability and cell identity if not washed off properly during thawing.
  c. To quickly thaw the cells, place the bottom of the vial in a water bath set at 37°C and swirl in a circular motion to ensure even thawing.
  d. Continue until only a small ice ball (∼3 mm diameter) remains in the tube (∼1 min). Transfer to a tissue culture hood.
    **CRITICAL:** Cell thawing should be performed rapidly and without delay to ensure maximum cell viability.
  e. Pre-warm a 50 mL aliquot of ESC basal media to 37°C, in a water bath (∼10 min). Add 1 mL pre-warmed ESC basal media to the vial and transfer the contents to the prepared 15 mL Falcon tube.
  f. Pellet cells by centrifugation at 1.2 **×** 10^3^ rcf, 4 min at room temperature (RT; 18°C–23°C).
  g. Aspirate supernatant, carefully avoiding the pelleted cells.
  h. Resuspend pelleted cells with 1 mL ESC complete media. Ensure sufficient resuspension by repeatedly pipetting the whole volume up and down carefully to avoid bubbles.
  i. Transfer the resuspended cells to an appropriate number of wells in a tissue-culture treated 6-well plate (approximately 1 **×** 10^4^ cells/well). We use plasma-treated polystyrene plates for tissue-culture, sourced commercially (e.g., ThermoFisher, cat. num. 10578911). Coating with 0.1% Gelatin (e.g., Merk, cat. num. ES-006-B) for 1 h at RT can also be used to improve cell adherence.
  j. Top up each well to a total of 2 mL ESC complete media (see materials & equipment for composition). Ensure homogeneous distribution of cells by firmly but smoothly agitating the plate in a cross-like motion.
  k. Check cell dispersion of each well using an inverted microscope with transmitted light and a low magnification objective (e.g., 4**×**, 10**×**). Cells should be small, single and homogeneously distributed throughout the well.
  l. Place plates in an incubator, stabilized at 37°C and 5% CO_2_.

2. Culture ESCs for 3 days, or until the formation of mid-sized, dome-shaped colonies:
  a. Daily, check cells under a microscope to monitor cell growth.
  b. After 24 h culture, and then every other day, aspirate ESC media and replenish with 2 mL pre-warmed ESC complete media.
  c. Optimally, most ESC colonies will be mid-sized (50–100 μm in diameter, estimated under a calibrated microscope with a reference scale-bar), dome-shaped and with refringent edges. Flat colonies, or colonies with “spiky”/irregular edges indicate cell differentiation (Figure 1). Mid-sized colonies can be used for experiments or passaged.

**CRITICAL:** Mycoplasma testing should be carried out routinely (*e.g.* every other week) (Nikfarjam and Farzaneh, 2012). Commercial kits such as “PCR Mycoplasma Test Kit I/C” (Promocell, cat. num. PK-CA91-1096) can be used. This is critical to maintain batch-to-batch consistency in the ESC culture.

3. Passage ESCs:
  a. To passage ESCs, aspirate culture media and rinse once with 2 mL PBS.
  b. Add 0.5 mL 0.25% Trypsin-EDTA/well and return the plate to the incubator. Alternatively, lower Trypsin-EDTA concentrations (e.g., 0.05%), or other cell lifting regents (e.g., Accutase) could be used. For those, the protocol, including incubation time and volume will need to be optimized.
  c. Incubate for <4 min at 37°C, 5% CO_2_ until cells are detached.
    **CRITICAL:** Do not incubate cells with 0.25% Trypsin-EDTA for longer than 5 minutes to minimize the possibility of cleaving the receptors of interest. Troubleshooting 2
  d. With a 5 mL serological pipette, use 2 mL (i.e., >3**×** the volume of 0.25% Trypsin-EDTA) ESC basal media to resuspend the cells. Transfer cell suspension to a 15 mL Falcon tube. Optionally, use a 1 mL pipette to dissociate colonies to a single cell suspension.
  e. Pellet cells by centrifugation at 1.2 **×** 10^3^ rcf, 4 min at RT.
  f. Aspirate supernatant, carefully avoiding the pelleted cells.
  g. Resuspend pelleted cells in 1 mL ESC complete media by repeatedly pipetting up and down carefully to avoid bubbles.
  h. Using a glass hemocytometer (i.e., Neubauer counting chamber), estimate the number of cells in the suspension. Briefly, a sample of the cell suspension is diluted in 0.4% Trypan Blue (e.g., 1:5 dilution, final concentration, 0.32% Trypan Blue). 10 μL of the diluted sample are carefully added to the counting chamber by capillary action. Under a microscope, using a 10**×** objective, unstained cells from each corner quadrant are counted, and the number of cells/quadrant is averaged. Cell density in the solution can be calculated by multiplying the averaged number by 10^4^ and correcting by the dilution factor (e.g., multiply by 5).
  i. In a 15 mL Falcon tube, mix the appropriate volumes of cell suspension (enough to reach approximately 1 **×** 10^4^ cells/well) and pre-warmed ESC complete media (e.g., 2 mL/well).
  j. Transfer the mix from the previous step to an appropriate number of wells in a tissue culture-treated 6-well plate (described in step 1i).
  k. Return plate to the incubator and culture at 37°C, 5% CO_2_. Cells will be ready for experiments after 1–2 passages.

### ESC transition to an early primed ESC stage

**Timing: 4 days**

This protocol describes the transition of ESCs to a primed ESC stage, as used in our experiments, based on the protocol published by ten Berge et al., 2011. ESCs expressing fluorescent constructs (e.g., GCaMP6s, Ftractin-mRuby3) can also be used. Notably, recent published protocols describe alternative and more stage-specific early primed ESC states that could also be used for the experiment described below (Kinoshita et al., 2020; Neagu et al., 2020). Additional troubleshooting may be required when using these cells.

4. Set up ESCs for pluripotency stage transition:
  a. Starting from a culture of ESCs (as described in steps 1–3), prepare a single cell suspension (following steps 3a-h).
  b. In a 15 mL Falcon tube, mix the appropriate volumes of cell suspension (enough to reach approximately 1 **×** 10^4^ cells/well) and pre-warmed ESC basal media (e.g., 2 mL/well). Add a final 2 μM IWP2 (Milteny, cat. num. 130-105-335) to the media and mix by pipetting.
  c. Transfer the mix from the previous step to an appropriate number of wells in a 6-well plate.
  d. Return plate to the incubator and culture at 37°C, 5% CO_2_.
5. Change the media and assess pluripotency stage transition:
  a. Every 24 h, aspirate the media and add 2 mL/well pre-warmed ESC basal media with 2 μM IWP2.
  b. Continue culturing the cells for 3 days, changing the media daily and assessing colony morphology under an inverted microscope with transmitted light. Primed ESC colonies will appear as flat colonies with “spiky” edges. In our experiments we used cells after 3 days of pluripotency transition (Figure 1). Additional characterization can be performed by qPCR, immunofluorescence or alkaline phosphatase staining (AP) to verify naïve to early-primed ESC transition (e.g., upregulation of Otx2 or Fgf5, downregulation of Nanog or Klf4, reduced AP staining in primed ESCs (Junyent et al., 2020; ten Berge et al., 2011; Ying et al., 2008)

**CRITICAL:** Mycoplasma testing should be carried out routinely (Nikfarjam and Farzaneh, 2012). Commercial kits such as “PCR Mycoplasma Test Kit I/C” (Promocell, cat. num. PK-CA91-1096) can be used. This is critical to maintain batch-to-batch consistency in the primed ESC culture.

### Culture of mouse trophoblast stem cells (TSCs)

**Timing: 7 days to several weeks**

This protocol describes the thawing, culture and passaging of TSCs. The TSCs used in our experiment were derived by the Rossant Lab (Tanaka et al., 1998), and constitutively express GFP. Using low-passage TSCs (below 30 passages) is recommended, while routinely confirming the expression of throphoblast stem cell markers (Tanaka et al., 1998). As part of the protocol, a layer of mitotically inactivated mouse embryonic fibroblasts (MEFs) will be prepared. Low-passage mitotically inactivated MEFs (passage 3) are sourced from commercial vendors (R&D Systems, cat. num. PSC001). During passaging, MEFs will be separated from TSCs by selective adhesion.

6. Prepare a layer of mitotically inactivated MEFs:
  a. Transfer a vial of mitotically inactivated MEFs (R&D Systems, cat. num. PSC001) from a long-term storage unit (e.g., LN2 tank) to a polystyrene box containing dry-ice pellets.
  b. In a tissue culture hood, prepare a 15 mL Falcon tube with 4 mL MEF media (see materials & equipment for composition).
  c. To quickly thaw the cells, place the bottom of the vial in a water bath, set at 37°C and swirl the tube in a circular motion to ensure even thawing.
  d. Continue thawing until only a small ice ball (∼3 mm diameter) remains in the tube (∼1 min). Transfer to a tissue culture hood.
    **CRITICAL:** Cell thawing should be performed rapidly and without delay to ensure maximum cell viability.
  e. Add 1 mL pre-warmed MEF media to the vial and transfer the contents to the prepared 15 mL Falcon tube.
  f. Pellet cells by centrifugation at 0.8 **×** 10^3^ rcf, 4 min at RT.
  g. Aspirate supernatant, carefully avoiding the pelleted cells.
  h. Resuspend pelleted cells with 1 mL MEF media. Ensure sufficient resuspension by repeatedly pipetting the whole volume up and down.
  i. Transfer the resuspended cells to an appropriate number of wells in a tissue-culture treated 6-well plate (approximately 2.5 **×** 10^5^ cells/well). Plates do not require gelatin coating before seeding the cells. Prepare several wells.
  j. Top up each well to a total of 2 mL MEF media. Ensure homogeneous distribution of cells by firmly but smoothly agitating the plate in a cross-like motion.
  k. Check cell dispersion on each well using an inverted microscope with transmitted light and a low magnification objective (e.g., 4**×**, 10**×**). Cells should be large, single and homogeneously distributed throughout the well.
  l. Place plates in an incubator, stabilized at 37°C and 5% CO_2_. Incubate for 24 h before proceeding to the next step. MEF-coated plates should be used within a week of seeding, and the media should be changed every other day.

7. Thaw a vial of cryopreserved TSCs:
  a. Prepare the recipient MEF-coated wells by aspirating the media, rinsing twice with PBS and adding 1 mL pre-warmed TSC media (see materials & equipment for composition) at least 15 min before thawing TSCs. Return plates to the incubator at 37°C and 5% CO_2_.
  b. Transfer the vial from a long-term storage unit (e.g., LN2 tank) to a polystyrene box containing dry-ice pellets.
  c. In a tissue culture hood, prepare a 15 mL Falcon tube with 4 mL TSC media.
  d. To quickly thaw the cells, place the bottom of the vial in a water bath, set at 37°C and swirl the tube in circular motions to ensure even thawing.
  e. Continue thawing until only a small ice ball (∼3 mm diameter) remains in the tube (∼1 min). Transfer to a tissue culture hood.
    **CRITICAL:** Cell thawing should be performed rapidly and without delay to ensure maximum cell viability.
  f. Add 1 mL pre-warmed TSC basal media to the vial and transfer the contents to the prepared 15 mL Falcon tube.
  g. Pellet cells by centrifugation at 1.2 **×** 10^3^ rcf, 4 min at RT.
  h. Aspirate supernatant, carefully avoiding the pelleted cells.
  i. Resuspend pelleted cells with 1 mL TSC media.
    **CRITICAL:** Unlike the resuspension of other cells, avoid repeated pipetting to maintain small TSC clusters (2–5 cells/cluster). Use a 1 mL pipette and gently pipette up and down twice, while avoiding generating bubbles.
  j. Aspirate TSC media from the MEF coated wells.
  k. Transfer the resuspended cells to an appropriate number of MEF coated wells in a 6-well plate.
  l. Top up each well to a total of 2 mL TSC media. Ensure homogeneous distribution of cells by firmly but smoothly agitating the plate in a cross-like motion.
  m. Check cell dispersion on each well using an inverted microscope with transmitted light and a low magnification objective (e.g., 4**×**, 10**×**). Cells should be small clusters, homogeneously distributed throughout the well.
  n. Place plates in an incubator, stabilized at 37°C and 5% CO_2_.

8. Culture TSCs for 5 days, until the formation of large, flat TSC colonies:
  a. Daily, monitor cell growth under an inverted microscope.
  b. 24 h after seeding, and then every other day, aspirate media and replenish with 2 mL/well pre-warmed TSC media.
  c. Culture cells for approximately 5 days or until the formation of large, round colonies with refringent edges in a layer of MEFs. Since these TSCs express GFP, TSC colonies can be distinguished using fluorescent light (Figure 1).

**CRITICAL:** Mycoplasma testing should be carried out routinely (Nikfarjam and Farzaneh, 2012). Commercial kits such as “PCR Mycoplasma Test Kit I/C” (Promocell, cat. num. PK-CA91-1096) can be used. This is critical to maintain batch-to-batch consistency in the TSC culture.

9. Passage TSCs:
  a. 24 h before passaging, prepare an appropriate number of MEF-coated wells in a 6-well plate (following step 6).
  b. To passage TSCs, aspirate TSC culture media and rinse once with PBS.
  c. Add 0.5 mL 0.05% Trypsin-EDTA to a well of TSCs and return to incubator.
  d. Incubate for <4 min at 37°C, 5% CO_2_ until cells are detached. Aim to obtain a suspension of small TSC clusters (2–5 cells per cluster) mixed with single MEFs.
    **CRITICAL:** Do not incubate cells with 0.05% Trypsin-EDTA for longer than 5 minutes to minimize the possibility of cleaving the receptors of interest. Troubleshooting 2
  e. With a 5 mL serological pipette, use 2 mL TSC media to resuspend the cells. Transfer cell suspension to a 15 mL Falcon tube.
  f. Pellet cells by centrifugation at 1.2 **×** 10^3^ rcf, 4 min at RT.
  g. Aspirate supernatant, carefully avoiding the pelleted cells.
  h. Resuspend pelleted cells in 1 mL TSC media by repeatedly pipetting up and down.
  i. To separate MEFs from TSCs, transfer the cell suspension to a clean 6-well plate and return to the incubator.
  j. Incubate for ∼15 min at 37°C, 5% CO_2_ to allow MEFs to attach.
  k. Carefully, pipette out the TSC-containing supernatant without disturbing the attached MEFs. Transfer to a 15 mL Falcon tube.
  l. Pellet cells by centrifugation at 1.2 **×** 10^3^ rcf, 4 min at RT.
  m. Aspirate supernatant, carefully avoiding the pelleted cells.
  n. Resuspend pelleted cells in 1 mL TSC media by briefly pipetting up and down.
  o. Using a glass hemocytometer, estimate the concentration of cell clusters (2–3 cells/cluster) in the suspension. Follow step 3 h., but count small cell clusters (typically 3–5 cells) instead of single cells.
  p. In a 15 mL Falcon tube, mix the appropriate volume of cell suspension (enough to reach approximately 2 **×** 10^4^ clusters/well) with the appropriate volume of pre-warmed TSC media (e.g., 2 mL/well).
  q. Transfer the mix from the previous step to an appropriate number of MEF-coated wells in a 6-well plate.
  r. Return plate to the incubator and culture at 37°C, 5% CO_2_

### Preparation of ESC lines expressing the Ca^2+^ sensor GCaMP6s or the F-actin reporter Ftractin-mRuby3.

**Timing: 2 weeks**

This protocol describes the production of GCaMP6s or Ftractin-mRuby3 lentiviruses in HEK293T cells, the transduction of ESCs, and the purification of GCaMP6s-expressing or Ftractin-mRuby3-expressing ESC lines. Low-passage HEK293T cells (<30 passages) should preferentially be used, as they will result in higher lentivirus yields.

10. Thaw a vial of HEK293T cells, following the protocol described for MEFs (step 6)
  a. Culture HEK293T cells in 10 cm^2^ plates until sub-confluent (∼80%), changing the media (HEK293T media is the same as MEF media, see materials & equipment for composition) every other day.
11. Prepare HEK293T cells for virus generation:
  a. Aspirate culture media from a plate of HEK293T cells. Carefully, rinse once with PBS.
  b. Add an appropriate volume of 0.25% Trypsin-EDTA (approx. 2 mL/plate) and return to the incubator.
  c. Incubate for <4 min at 37°C, 5% CO_2_ until cells are detached. Aim to generate a single cell suspension.
  d. With a 10 mL serological pipette, use 5 mL HEK293T media to resuspend the cells. Transfer cell suspension to a 15 mL Falcon tube.
  e. Pellet cells by centrifugation at 1.2 **×** 10^3^ rcf, 4 min at RT.
  f. Aspirate supernatant, carefully avoiding the pelleted cells.
  g. Resuspend pelleted cells in 1 mL HEK293T media by repeatedly pipetting up and down.
  h. Using a glass hemocytometer, estimate the concentration of cells in the suspension.
  i. In a 15 mL Falcon tube, mix the appropriate volume of cell suspension (enough to reach approximately 3.8 **×** 10^6^ cells/10 cm plate) with the appropriate volume of pre-warmed HEK293T media (e.g., 7 mL/plate).
  j. Transfer the mix from the previous step to a 10 cm tissue-culture treated plate and return to the incubator.
  k. Incubate cells for 24 h at 37°C, 5% CO_2_

12. Generate lentivirus:
  a. 24 h after seeding, aspirate HEK293T culture media and rinse once with PBS.
  b. Add 7 mL pre-warmed HEK293T media without Penicillin-Streptomycin.
  c. Return plate to the incubator and incubate for >3 h at 37°C, 5% CO_2_
  d. To transfect HEK293T cells with the plasmids required for lentivirus preparation, prepare a JetPrime transfection mix in a 1.5 mL Eppendorf tube, as follows:
    **Table:** 

JetPrime transfection mix		
Order of mixing	Component	Amount
1^st^	JetPrime Buffer	500 μL
2^nd^	GCaMP6s plasmid or Ftractin-mRuby3 plasmid	5 μg
3^rd^	psPAX2 plasmid	3.2 μg
4^th^	PMD2.G plasmid	1.6 μg

**Quickly vortex the mix (2 s)**		

5^th^	JetPrime reagent	20 uL

Use immediately after incubation, do not store.
  e. Immediately after JetPrime reagent addition, vortex the transfection mix continuously and vigorously for 10 s.
  f. Incubate the transfection mix still, at RT for 10 min.
  g. Add the transfection mix to a plate of HEK293T cells, dropwise.
    **CRITICAL:** Add transfection mix dropwise, distributed homogeneously throughout the plate. To ensure homogeneous mixing, gently agitate the plate on a flat surface, following a cross-like pattern.
  h. Return plate to the incubator and incubate for 24 h at 37°C, 5% CO_2_.
    **CRITICAL:** From this point onwards transfected HEK293T cells are producing lentiviruses. Handle these cells, and any materials that may have been in contact with them, carefully. Follow institution-specific guidelines in how to treat and discard lentivirus-related material. We recommend incubating any material that has contacted lentivirus (*i.e.,* pipette tips, media, plates, etc.) with a 2% bleach solution for 10 min.
  i. After 24 h, aspirate and discard culture media, rinse once with PBS and replenish with 7 mL media. Return plate to the incubator and incubate for 24 h at 37°C, 5% CO_2_
  j. After 24 h, **collect** culture media in a 50 mL Falcon tube. Do not rinse with PBS. Replenish with 7 mL media. Return plate to the incubator and incubate for 24 h at 37°C, 5% CO_2_. Store lentivirus-containing media at 4°C.
  k. After 24 h, **collect** culture media. Discard plate. Pool lentivirus-containing media with the media previously collected in step j. Filter media with a 0.45 μm filter.
  l. To concentrate lentivirus, add 4.6 mL Lenti-X concentrator to 14 mL lentivirus-containing media (i.e., 1:3 Lenti-X:media), mix thoroughly by pipetting and incubate at 4°C, overnight (12–20 h).
  m. Concentrate lentiviruses by centrifugation at 1.5 **×**10^3^ rcf for 45 min at 4°C.
  n. Return to the tissue culture hood and carefully aspirate the supernatant without disturbing the pellet.
  o. Resuspend pellet with 2 mL ESC basal media. Aliquot as single-use volumes (e.g., 250 μL for 6 well plate) and use immediately (following step 13) or store at –80°C (up to 2 years, although transduction efficiency might decay with time). Lentivirus tilter can be quantified by transducing a control cell line (e.g., HEK293T) with serial dilutions of the virus stock and measuring the number of GCaMP6s- or Ftractin-mRuby3 expressing-cells by FACS (following steps 13 and 14).

13. Transduce ESCs with GCaMP6s or Ftractin-mRuby3 lentivirus:
  a. Prepare a sub-confluent (approx. 3 days after seeding, mid-sized colonies) well of ESCs following step 3.
  b. On the day of transduction, aspirate culture media and replenish with 2 mL pre-warmed ESC complete media. Return to the incubator for >1 h.
  c. Thaw a vial of GCaMP6s or Ftractin-mRuby3 lentiviruses at RT. Add content dropwise to the well of ESCs.
    **CRITICAL:** Optimization of the amount of virus used for transduction might be required to ensure optimal cell fitness. To do so, different concentrations/volumes of virus should be used, and cell growth and ESC characteristics should be verified (described in step 2c).
  d. Return plate to the incubator and incubate for 48 h at 37°C, 5% CO_2_. After transduction, cells should maintain normal morphology and ESC qualities, analyzed as described in step 2c.

14. Purify a GCaMP6s-expressing or Ftractin-mRuby3-expressing ESC line.
  a. An ESC line expressing GCaMP6s or Ftractin-mRuby3 can be purified by Fluorescence Activated Cell Sorting (FACS). Briefly, a single cell suspension is prepared following step 3a-f. Then, cells are resuspended in 250 μL FACS buffer (3% FBS in PBS), with or without DAPI (0.5 μg/mL). Non-transduced cells are used as control and prepared in the same way.
  b. Cell sorting is performed using a FACS (e.g., FACSAria III). DAPI-negative, GFP-positive (GCaMP6s) or mRuby3-positive (Ftractin-mRuby3) ESCs are sorted to a tube containing 3 mL ESC complete media.
  c. Following sorting, cells are seeded in 6-well plates and cultured as described in steps 2–3. GCaMP6s or Ftractin-mRuby3 expression can be verified using an inverted microscope with fluorescent illumination.
  d. If required, a second FACS-sorting round can ensure homogeneous GCaMP6s or Ftractin-mRuby3 expression levels. Troubleshooting 3

### Culture of SuperTOPFlash Wnt/β-catenin pathway reporter LS/L cells (LSLs)

**Timing: 3 days to several weeks**

This protocol briefly describes how to culture LSLs: L-cells stably expressing the Wnt reporter SuperTopFlash (Fuerer and Nusse, 2010). LSL culture follows the same steps and uses the same media as MEF and HEK293T culture (above).

15. Thaw a vial of cryopreserved LSLs, as described for MEFs (step 6), and culture cells until sub-confluent (∼80%):
  a. Daily, check cells under a microscope to monitor cell growth.
  b. After 24 h culture, and then every other day, aspirate media and replenish with 7 mL pre-warmed LSL media.

### Preparation of Wnt3a-beads and control beads and Wnt/β-catenin pathway activation assay

**Timing: 2 days**

This protocol briefly describes the preparation of Wnt3a-beads, and the steps followed to test their biological activity. For a comprehensive step-by-step protocol, reagents used and their preparation, please refer to (Habib et al., 2013; Lowndes et al., 2017).

16. Microbead surface activation:
  a. The carboxylic acid surface of Dynabeads (30 μg, resuspended in 25 mM MES buffer) (2-(N-morpholino)ethanesulfonic acid in water, pH 5) is functionalized by incubation with 50 mg/mL of both EDC (1-Ethyl-3-(3-dimethylaminopropyl)carbodiimide) and NHS (N-hydroxysuccinimide) in MES buffer, for 30 min at RT (with slow rotation, 30 rpm).
  b. For transferring beads between solutions, or for washes, microbeads are immobilized with a magnet placed against the wall of the tube for a 1 min.
17. Immobilization of Wnt proteins onto microbeads.
  a. Following three washes (5 min each) in MES, functionalized microbeads are incubated with a mixture of 20 μL recombinant Wnt3a (40 μg/mL stock) with a 5**×** volume of MES buffer for 1 h at RT (with slow rotation). To prepare other control beads (e.g., Wnt5a-beads, BSA-beads...), an equivalent concentration of protein should be added to the functionalized microbeads and the same protocol should be followed.
  b. After Wnt immobilization, beads are washed in PBS 3 times (5 min each) and resuspended in 300 μL ESC basal media for quenching.
    ***Optional:*** to produce iWnt3a-beads (inactivated Wnt3a controls), inactivate the disulfide bonds of Wnt3a proteins through resuspending pelleted beads in 20 mM DTT (Dithiothreitol) in water and incubating at 37°C for 45 min. After inactivation, follow step 17 b.
  c. Wnt3a-beads and inactivated Wnt3a-beads can be stored at 4°C for up to 2 weeks.

18. Assaying Wnt3a-bead or control bead activity with LSL Luciferase assay
  a. The capacity of Wnt3a-beads to activate the Wnt/β-catenin pathway is tested using LSLs. For each condition tested, 5 **×** 10^4^ LSL cells in 100 μL media are seeded in a well of a 96-well plate, in triplicate, and incubated until cell attachment (4–6 h). Then, diluted into a further 100 μL of media, 2 μg Wnt3a-beads or inactivated Wnt3a-beads/well, 200 ng/mL solubilized Wnt3a or an equivalent volume of control solution are added to the wells.
    **CRITICAL:** To ensure homogeneous distribution of the Wnt-beads, triturate the Wnt-bead stock tube thoroughly before adding it to the cells. This is done by pipetting up and down >100 times. We also recommend doing this step in two parts: mixing the Wnt-bead stock thoroughly before adding it to a 1.5 mL Eppendorf tube with 100 μL LSL media, mixing again and then adding the diluted Wnt-beads to the cells. Skipping this step will result in large Wnt-bead aggregates, inefficient Wnt/β-catenin pathway activation, and inaccurate readings.
  b. LSLs are incubated overnight (>16 h) at 37°C, 5% CO_2_.
  c. To measure Wnt-mediated Luciferase production, LSLs are lysed with passive lysis buffer and cell lysates are evaluated with the Dual-Light System assay. Luciferase activity is determined using a Glomax-Multi detection system (Promega).
  d. If successfully immobilized, Wnt3a-beads should activate Luciferase production in LSLs by at least 100-fold over the negative control (Lowndes et al., 2017). Troubleshooting 5.

## Key resources table

**Table:** 

REAGENT or RESOURCE	SOURCE	IDENTIFIER
**Chemicals, peptides, and recombinant proteins**		

Recombinant Mouse Wnt-3a Protein	R&D systems	Cat#1324-WN-010
Mouse LIF	Miltenyi Biotec	Cat#130-095-778
StemMACS™ CHIR99021	Miltenyi Biotec	Cat#130-106-539
StemMACS™ PD0325901	Miltenyi Biotec	Cat#130-103-923
Recombinant mouse FGF-4 Protein	R&D systems	Cat#5846-F4-025
Heparin sodium salt from porcine intestinal mucosa	Sigma	Cat#H3149-10KU
IWP2	Tocris	Cat#3533
Dithiothreitol (DTT)	Life Technologies	Cat#P2325
Bovine Serum Albumin	Thermo Fisher Scientific	Cat#155561020
Kainic Acid monohydrate 98%	Thermo Fisher Scientific	Cat#15467999
Dymethyl Sulfoxide (DMSO)	Sigma	Cat#D5879
CNQX	Sigma	Cat#C127
Fetal Bovine Serum	Sigma	Cat#F7524
2-mercaptoethanol	Sigma	Cat#21985-023
Dulbecco's Modified Eagle Medium (DMEM)	Thermo Fisher Scientific	Cat#11965092
Glutamax	Sigma	Cat#G7513
Penicillin-Streptomycin	Sigma	Cat#P4458
Fetal Bovine Serum - ESC qualified	Millipore	Cat#ES-009-B
RMPI1640	Gibco	Cat#11875085
Na-Pyruvate	Gibco	Cat#11360070
Advanced DMEM/F12	Gibco	Cat#12634 028
Phosphate Buffered Saline	Sigma	Cat#D8662
Trypsin-EDTA (0.25%)	Thermo Fisher Scientific	Cat#25200056
Trypan Blue solution (0.4%)	Sigma	Cat#T8154
Dynabeads™ M-270 Carboxylic Acid	Thermo Fisher Scientific	Cat#14305D
MES (2-(N-morpholino)ethanesulfonic acid)	Merck	Cat#M3671
EDC (1-Ethyl-3-(3-dimethylaminopropyl)carbodiimide)	Merck	Cat#341006
NHS (N-hydroxysuccinimide)	Merck	Cat#130672

**Critical commercial assays**		

Dual-Light™ Luciferase & β-Galactosidase Reporter Gene Assay System	Thermo Fisher Scientific	Cat#T1003
jetPRIME®	Polyplus transfection	Cat#114-07

**Experimental models: Cell lines**		

W4 (129S6/SvEvTac) mouse embryonic stem cells	(Auerbach et al., 2000)	RRID:CVCL_Y634
Mouse Trophoblast Stem Cells	Tanaka et al. (1998)	N/A
Irradiated Mouse Embryonic Fibroblasts	R&D systems	Cat#PSC001
L Cells	ATCC	Cat#CRL-2648
HEK-293	ATCC	Cat#CRL-1573

**Recombinant DNA**		

pLV-Ftractin-mRuby3-p2A-mTurquoise-MLC-IRES-Blast	Tobias Meyer lab (Hayer et al., 2016)	Addgene: #85146
pGP-CMV-GCaMP6s	Douglas Kim lab (Chen et al., 2013)	Addgene: #40753
psPAX2	Didier Trono	Addgene: #12260, RRID:Addgene_12260
pMD2.G	Didier Trono	Addgene: #12259, RRID:Addgene_12259
pSuperTOPFlash-Luciferase reporter	Randall Moon (Veeman et al., 2003)	Addgene: #12456

**Software and algorithms**		

Fiji	ImageJ	RRID:SCR_002285
NIS Elements	Nikon	RRID:SCR_014329
Prism	GraphPad	RRID:SCR_002798

**Other**		

Corning 96-well Clear Bottom Black Polystyrene Microplates	Thermo Fisher Scientific	Cat#07-200-565
6-Well Tissue Culture Plates	Thermo Fisher Scientific	Cat#10578911
μ-slide 8 well ibiTreat slide	IBIDI	Cat#80826
Eclipse Ti inverted, equipped with a Yokogawa CSU-1 disk head and an Andor Neo sCMOS camera	Nikon	N/A
Neubauer Improved Hemocytometer Counting Chamber	Hawksley	Cat#AC1000
BD FACSAria III Cell Sorter	BD Biosciences	N/A

## Materials and equipment

**Table:** LIF solution (1 **×** 10^7^ U/uL)

Reagent	Final concentration	Amount
Mouse Leukemia Inhibitory Factor (LIF)	1 **×** 10^7^ U/mL	25 μg
1% BSA-PBS (w/v)	n/a	250 μL
**Total**	**n/a**	**250 μL**

Aliquot to small volumes and store up to 1 year at −20°C.

**Table:** ESC basal media

Reagent	Final concentration	Amount
Advanced DMEM/F12	1**×**	440 mL
FBS – ES Cell Qualified (not heat inactivated).	10% (v/v)	50 mL
Penicillin-Streptomycin	1% (v/v)	5 mL
2-mercaptoethanol	50 μM	454 μL
Glutamax	2 mM	5 mL
Mouse Leukemia Inhibitory Factor (LIF)	1 **×** 10^3^ U/mL	50 μL
**Total**	**n/a**	**500 mL**

Filter sterile with a 0.22 μm filter. Store up to 4 weeks at 4°C.

**Table:** CHIR99021 solution (10 mM)

Reagent	Final concentration	Amount
CHIR99021	10 mM	2 mg
DMSO	n/a	429.8 μL
**Total**	**n/a**	**429.8 μL**

Aliquot to single-use volumes (e.g., <100 μL) and store up to 1 year at –20°C.

**Table:** PD0325901 solution (10 mM)

Reagent	Final concentration	Amount
PD0325901	10 mM	2 mg
DMSO	n/a	414.8 μL
**Total**	**n/a**	**414.8 μL**

Aliquot to single-use volumes (e.g., <100 μL) and store up to 1 year at –20°C.

**Table:** IWP2 (5 mM)

Reagent	Final concentration	Amount
IWP2	5 mM	2 mg
DMSO	n/a	857.3 μL
**Total**	**n/a**	**857.3 μL**

Aliquot to single-use volumes (e.g., <10 μL) and store up to 1 year at –20°C.

**Table:** ESC complete media

Reagent	Final concentration	Amount
ESC basal media	1**×**	50 mL
CHIR99021	3 μM	15 μL
PD0325901	1 μM	5 μL
**Total**	**n/a**	**50 mL**

Store up to 2 weeks at 4°C.

**Table:** HEK293T/LSL/MEF media

Reagent	Final concentration	Amount
Dulbeco’s Modified Eagle Medium	1**×**	440 mL
FBS	10% (v/v)	50 mL
Glutamax	2 mM	5 mL
Penicillin-Streptomycin	1% (v/v)	5 mL
**Total**	**n/a**	**500 mL**

Store up to 4 weeks at 4°C

**Table:** FGF4 solution (100 μg/mL)

Reagent	Final concentration	Amount
FGF4	100 μg/mL	25 μg
0.1% BSA-PBS (w/v)	n/a	250 μL
**Total**	**n/a**	**250 μL**

Aliquot to small volumes and store up to 1 year at –20°C.

**Table:** Heparin solution (140 U/mL)

Reagent	Final concentration	Amount
Heparin	140 U/mL	1 **×** 10^4^ U
PBS	n/a	71.42 mL
**Total**	**n/a**	**71.42 mL**

Aliquot to small volumes and store up to 1 year at –20°C.

**Table:** TSC media

Reagent	Final concentration	Amount
RPMI1640	1**×**	385
FBS – ES Cell Qualified (not heat inactivated)	20% (v/v)	100 mL
Penicillin-Streptomycin	1% (v/v)	5 mL
2-mercaptoethanol	100 μM	900 μL
Glutamax	2 mM	5 mL
Na-Pyruvate	1 mM	5 mL
**Total**		**500 mL**
TSC basal media (above)	1**×**	50 mL
FGF4	25 ng/mL	12.5 μL
Heparin	1 μg/mL	25 μL
**Total**	**n/a**	**50 mL**

Filter sterile with a 0.22 μm filter and store without growth factors up to 4 weeks at 4°C. Store with growth factors for up to one week at 4°C

**Table:** MES Buffer

Reagent	Final concentration	Amount
MES ((2-(N-morpholino)ethanesulfonic acid)	25 mM	2.44 g
Deionized Water	n/a	500 mL
**Total**	**25 mM MES**	**500 mL**

Adjust to pH 5 with NaOH as required. Store up to 4 weeks at 4°C.

## Step-by-step method details

### Measuring cytoneme-mediated communication between ESCs or primed ESCs and TSCs

**Timing: 2 days**

This protocol describes how to set up and image a co-culture of ESCs or primed ESCs (expressing Ftractin-mRuby3) and TSCs to measure their interaction, using a spinning disk confocal microscope. Variations of this protocol include the pre-treatment of TSCs with Porcupine inhibitors IWP2 (inhibitor of Wnt secretion) (Chen et al., 2009) or Wnt-C59 (Cayman Chemicals, cat. num. 16644). We exemplify the use of IWP2 below. Alternatively, Kainate (100 μM), CNQX (10 μM) or other drug treatments can be added to the media after step 3 (below) (Junyent et al., 2020). Additionally, ESCs expressing GCaMP6s or other fluorescently tagged constructs, or ESCs knock-out (KO) for Wnt/β-catenin pathway components and ionotropic glutamatergic receptors have been used in our experiments (Junyent et al., 2021b). KO ESCs can be generated following published protocols (Ran et al., 2013). In these cases, construct-expressing or KO ESCs should be used from step 2 onwards.

1. Prepare a suspension of MEF-free TSCs
  ***Optional:*** 24 h before, add 2 μM IWP2 (or 1 μM Wnt-C59) to the media of one well of TSCs (IWP2-treated TSCs).
  a. Aspirate culture media from one well (or more) of TSCs, and rinse once with PBS.
  b. Add 0.5 mL 0.05% Trypsin-EDTA and return the plate to the incubator.
  c. Incubate for <4 min at 37°C, 5% CO_2_ until cells are detached. Aim to obtain a suspension of small TSC clusters (2–3 cells per cluster) mixed with single MEFs.
    **CRITICAL:** Do not incubate cells with 0.05% Trypsin-EDTA for longer than 5 minutes to minimize the possibility of cleaving the receptors of interest. Troubleshooting 2
  d. With a 5 mL serological pipette, use 2 mL TSC media to resuspend the cells. Transfer cell suspension to a 15 mL Falcon tube. For IWP2-treated TSCs, add 2 μM IWP2 to the media.
  e. Pellet cells by centrifugation at 1.2 **×** 10^3^ rcf, 4 min at RT.
  f. Aspirate supernatant, carefully avoiding the pelleted cells.
  g. Resuspend pelleted cells in 1 mL TSC media by repeatedly pipetting up and down.
  h. To separate MEFs from TSCs, transfer cell suspension to a clean 6-well plate and return to the incubator.
  i. Incubate for ∼15 min at 37°C, 5% CO_2_ to allow MEFs to attach.
  j. Carefully, pipette out the TSC-containing supernatant without disturbing the attached MEFs. Transfer to a 15 mL Falcon tube.
  k. Pellet cells by centrifugation at 1.2 **×** 10^3^ rcf, 4 min at RT.
  l. Aspirate supernatant, carefully avoiding the pelleted cells.
  m. Resuspend pelleted cells in 1 mL TSC media by briefly pipetting up and down.
  n. Using a glass hemocytometer, estimate the concentration of cell clusters in the suspension.

2. Prepare a suspension of ESCs or primed ESCs (see culture of mouse embryonic stem cells (ESCs) and ESC transition to an early primed ESC stage)
  a. Aspirate culture media from a well of ESCs or primed ESCs stably expressing Ftractin-mRuby3 (or expressing other fluorescently-tagged construct, or a KO ESC/primed ESC line), and rinse once with PBS.
  b. Add 0.5 mL 0.25% Trypsin-EDTA and return the plate to the incubator.
  c. Incubate for <4 min at 37°C, 5% CO_2_ until cells are detached.
    **CRITICAL:** Do not incubate cells with 0.25% Trypsin-EDTA for longer than 5 minutes to minimize the possibility of cleaving the receptors of interest. Troubleshooting 2
  d. With a 5 mL serological pipette, use 2 mL ESC basal media to resuspend the cells. Transfer cell suspension to a 15 mL Falcon tube.
  e. Pellet cells by centrifugation at 1.2 **×** 10^3^ rcf, 4 min at RT.
  f. Aspirate supernatant, carefully avoiding the pelleted cells.
  g. Resuspend pelleted cells in 1 mL ESC basal media by repeatedly pipetting the whole volume up and down.
  h. Using a glass hemocytometer, estimate the concentration of cell in the suspension.

3. Prepare ESC-TSC or primed ESC-TSC co-culture.
  a. Prepare a 1.5 mL Eppendorf tube, following:
    **Table:** ESC-TSC co-culture mix

Component	Concentration or volume
ESCs or primed ESCs	1,500 cells/well
TSCs	1,500 cells/well
ESC media	Total 200 μL/well
(*Optional*) IWP2	2 μM

    **CRITICAL:** Cell density should be optimized. In densities that are too low, ESC/primed ESC and TSCs will not be close enough to initiate the interaction. Densities that are too high will impede proper analysis as cells will be too close together and non-cytoneme mediated contacts (*e.g*. non-specific contact/collisions via cell motility) may occur (Figure 2) troubleshooting 1.
    **Figure 2:**
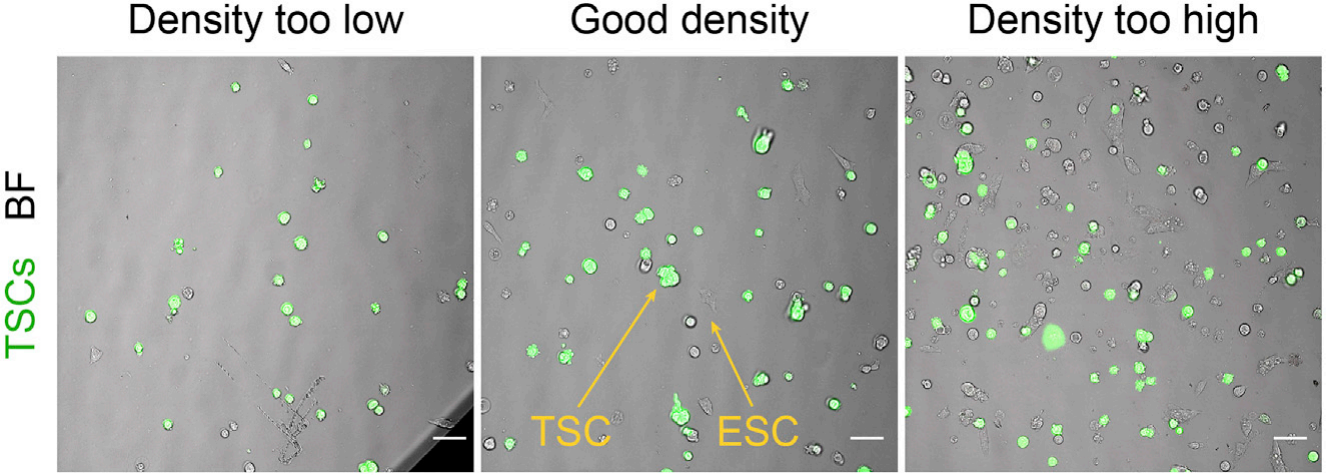
Representative images of the distribution of TSCs (green) and ESCs (imaged with brightfield, gray) Examples are shown for too low, good and too high seeding densities. Scale bars, 50 μm.
  b. Mix cells gently and transfer 200 μL mix to one well of a black-walled, clear-bottomed TC-treated, 96-well plate (e.g., Corning, cat. num. 3916). Return to the incubator.
  c. Incubate for 1 h at 37°C, 5% CO_2_ to allow cell attachment.

4. Set up imaging of ESC/pESC-TSC interaction in a spinning disk confocal microscope
  ***Optional:*** Transfer cells to a pre-warmed polystyrene box for transport between incubator and microscope.
  a. Pre-stabilize the incubation box of the spinning disk microscope at 37°C, 5% CO_2_
  b. Place the imaging 96-well plate in the motorized stage.
  c. Select the 20**×**/0.75 objective (or equivalent).
  d. Perform Kohler illumination adjustment of the transmitted light following standard procedures.
  e. Choose representative positions in which ESCs or primed ESCs and TSCs are closely located, but not contacting. Use the mRuby3 channel to visualize ESCs or primed ESCs and the thin structures in their cytonemes; use the GFP channel to localize TSCs. Choose ∼20 positions/well.
  f. Adjust laser intensities and exposure times to be as low as possible, to avoid phototoxicity (e.g., 30 ms for brightfield, 200 ms and 20% laser power for RFP-561 nm laser and GFP-488 nm laser with 4x bining in a Nikon Eclipse Ti Inverted microscope with a Yokogawa CSU-X1 disk head and Andor Neo sCMOS camera).
  g. Set up 3 z-positions to sample the full volume of the cell (approximately 3–5 μm apart). Center the Z-stack to the position with a higher resolution of the cytonemes.
  h. Set up the time-lapse imaging protocol: image each position every 10 min, for 12 h, for the following channels: brightfield, mRuby3 (RFP) and GFP.

***Optional:*** Image a one-cycle loop to check that the setup is correct.

**CRITICAL:** Choose enough positions to achieve a good representation of the ESC-TSC or primed ESC-TSC dynamics throughout the well, but few enough so a cycle of imaging can be performed within the 10 min interval.

5. Image ESC/primed ESC-TSC interaction overnight.
  a. Run automated imaging overnight (∼12 h).
  b. Perform reactivity analysis in the images obtained (see quantifying cytoneme reactivity to sources of Wnt).

### Measuring cytoneme-mediated recruitment of Wnt-beads by ESCs or primed ESCs

**Timing: 14 h**

This protocol describes how to set up and image a plate of ESCs or primed ESCs co-cultured with Wnt3a-beads or control-beads (e.g., iWnt3a-beads) to measure cytoneme-mediated localized Wnt recruitment, using a spinning disk confocal microscope. Variations of this protocol include adding Kainate (100 μM), CNQX (10 μM) or other drug treatments to the media (added after step 7, below). Additionally, cells expressing GCaMP6s or other fluorescently tagged constructs, or cells KO for Wnt/β-catenin pathway components and ionotropic glutamatergic receptors have been used in our experiments. In these cases, construct-expressing or KO ESCs/primed ESCs should be used from step 6 onwards.

6. Prepare a suspension of ESCs or primed ESCs
  a. From a 6-well plate culture of ESCs or primed ESCs stably expressing Ftractin-mRuby3, aspirate culture media and rinse once with PBS.
  b. Add 0.5 mL 0.25% Trypsin-EDTA and return the plate to the incubator.
  c. Incubate for ∼4 min at 37°C, 5% CO_2_ until cells are detached.
    **CRITICAL:** Do not incubate cells with 0.25% Trypsin-EDTA for longer than 5 minutes to minimize the possibility of cleaving the receptors of interest. Troubleshooting 2
  d. With a 5 mL serological pipette, use 2 mL ESC basal media to resuspend the cells. Transfer cell suspension to a 15 mL Falcon tube.
  e. Pellet cells by centrifugation at 1.2 **×** 10^3^ rcf for 4 min at RT.
  f. Aspirate supernatant, carefully avoiding the pelleted cells.
  g. Resuspend pelleted cells in 1 mL ESC basal media by repeatedly pipetting up and down.
  h. Using a glass hemocytometer, estimate the concentration of cell in the suspension.

7. Prepare ESCs/primed ESCs cultured with Wnt3a-beads or control beads.
  a. Dilute ESCs/primed ESCs to 3,000 cells per 100 μL in ESC basal media, for each well required.
  b. Add 0.3 μg of Wnt3a-beads or control beads per 3,000 cells.
  c. Add ESC basal media such that the final concentration is 3,000 cells and 0.3 μg beads per 200 μL.
    **CRITICAL:** Cell and bead density should be optimized. In low densities the Wnt-beads and the cells will be too far apart, and cells cannot initiate the interaction. Densities that are too high will impede proper analysis by increasing the non-specific (*i.e.,* not cytoneme-mediated) interactions between cells and Wnt-beads, as well as the contact between cells (Figure 3) Troubleshooting 1.
    **Figure 3:**
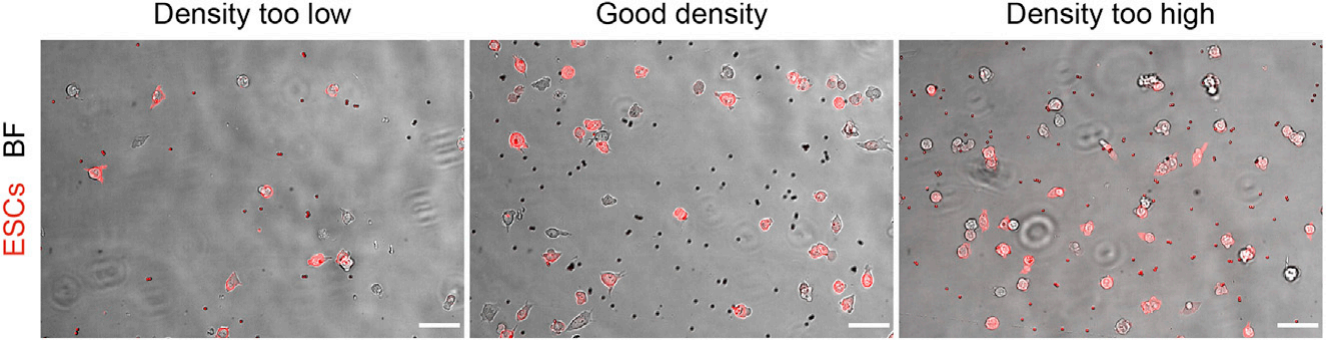
Representative images of the distribution of ESCs (red) and Wnt3a-beads (imaged with brightfield, gray) Examples are shown for too low, good and too high seeding densities. Scale bars, 50 μm.
  d. Transfer 200 μL of the mix to the wells of a black-walled, clear-bottomed TC-treated, 96-well plate (e.g., Corning, cat. Num. 3916).
  e. Return to the incubator and incubate for 1 h at 37°C, 5% CO_2_ to allow cell attachment.

8. Set up imaging of ESC/primed ESC-bead interactions in a spinning disk confocal microscope
  ***Optional:*** Transfer cells to a pre-warmed polystyrene box for transport to the microscope.
  a. Pre-stabilize the incubation box of the spinning disk microscope at 37°C, 5% CO_2_
  b. Place the cell-containing 96-well plate in the motorized stage.
  c. Select the 20**×**/0.75 objective (or equivalent).
  d. Perform Kohler illumination adjustment of the transmitted light following standard procedures.
  e. Choose representative positions in which ESCs and Wnt-beads or control-beads are closely located, but not already contacting. Use the mRuby3 channel to visualize ESCs or primed ESCs and the thin structures in their cytonemes. Choose ∼20 positions/well.
  f. Adjust laser intensities and exposure times to be as low as possible to avoid phototoxicity (e.g., 30 ms for brightfield, 200 ms and 20% laser power for RFP-561 nm laser with 4x bining in a Nikon Eclipse Ti Inverted microscope with a Yokogawa CSU-X1 disk head and Andor Neo sCMOS camera).
  g. Set up 3 z-positions covering the full volume of the cell (approximately 3–5 μm apart). Center the Z-stack to the position with better resolution of the cytonemes.
  h. Set up the time-lapse imaging protocol: image each position every 10 min, for 12 h, for brightfield and mRuby3 (RFP channel).

***Optional:*** Image a one-cycle loop to check that the setup is correct.

**CRITICAL:** Choose enough positions to achieve a good representation of the ESC-TSC dynamics throughout the well, but few enough so a cycle of imaging can be performed within the 10 min interval. In our experience, a maximum of 20–25 positions can be imaged at a time.

9. Imaging ESC/primed ESC-bead interactions
  a. Image for 12 h using the automated imaging protocol.
  b. Perform reactivity analysis in the images obtained (see quantifying cytoneme reactivity to sources of Wnt).

### Quantifying cytoneme reactivity to sources of Wnt (Wnt3a-beads or TSCs)

**Timing: 3–6 h**

**Figure 4:**
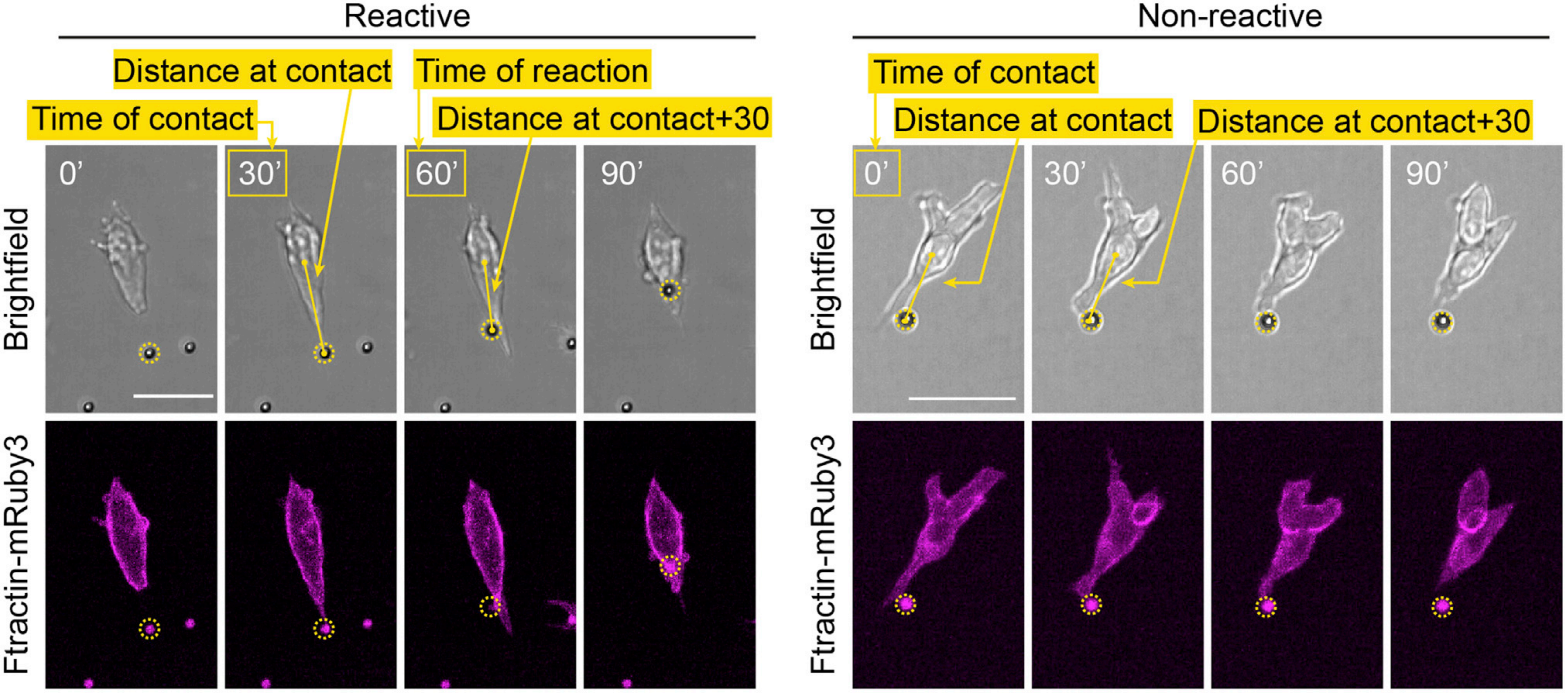
Representative frames of a time-course imaging showing ESCs (magenta) contacting Wnt3a-beads (black spheres, highlighted with dashed yellow circles) with a cytoneme Examples are shown for a “Reactive” (*left*) and a “Non-reactive” (*right*) interaction. Yellow lines or boxes indicate the measurements performed. Note that the distance between Wnt3a-bead and cell is reduced following contact in the “Reactive” interaction, but not in the “Non-reactive” one. Time is displayed in minutes. Scale bars, 25 μm.

**Figure 5:**
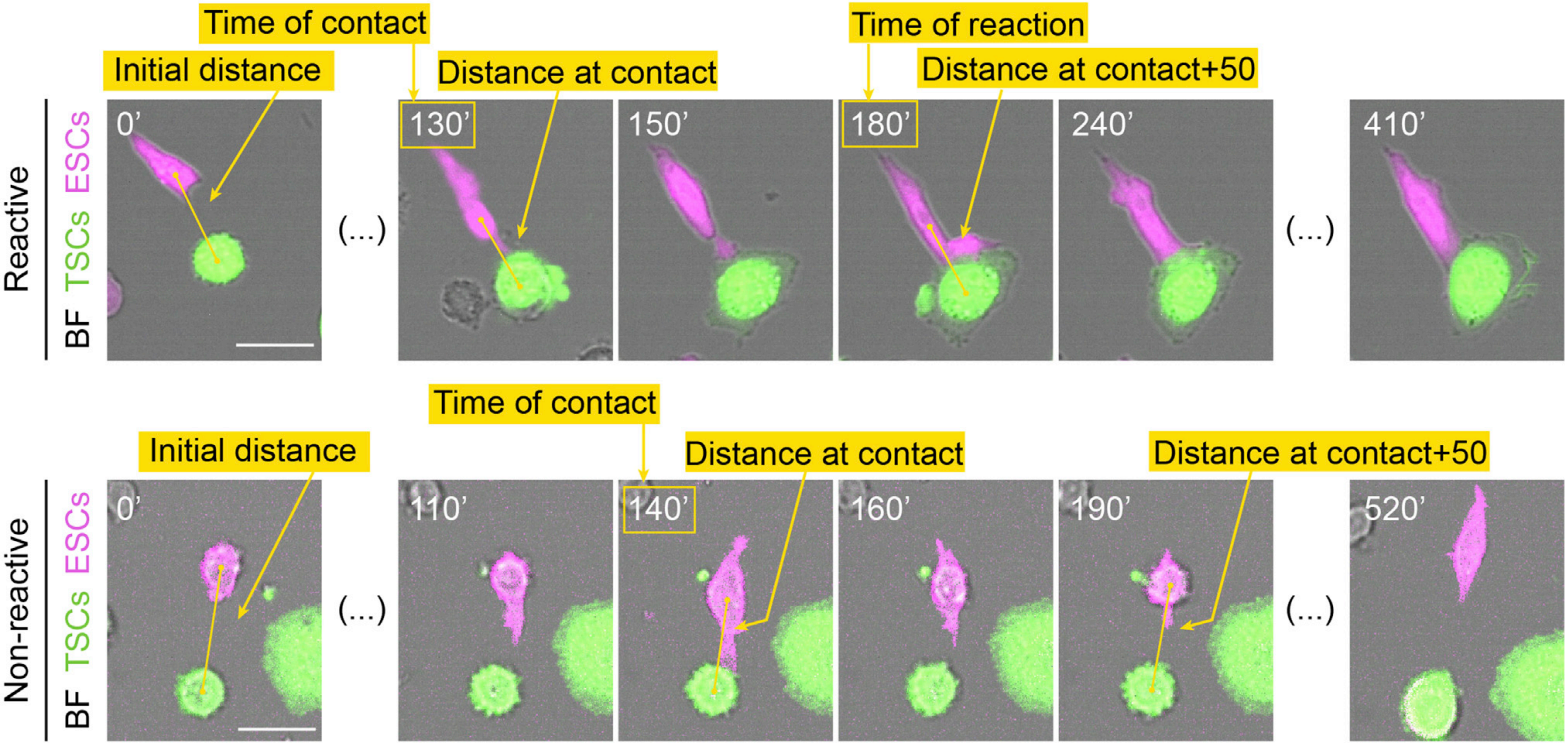
Representative frames of a time-course imaging showing ESCs (magenta) contacting TSCs (green) with a cytoneme Examples are shown for a “Reactive” (top) and a “Non-reactive” (*bottom*) interaction. The first frame shows the initial distance between the cells. The last frame shows retention of contact for “Reactive” interactions but not for “Non-reactive” interactions. Yellow lines or boxes indicate the measurements performed. Note that the distance between ESC and TSC is reduced following contact in the “Reactive” interaction, but not in the “Non-reactive” one. Time is displayed in minutes. Scale bars, 25 μm.

**Figure 6:**
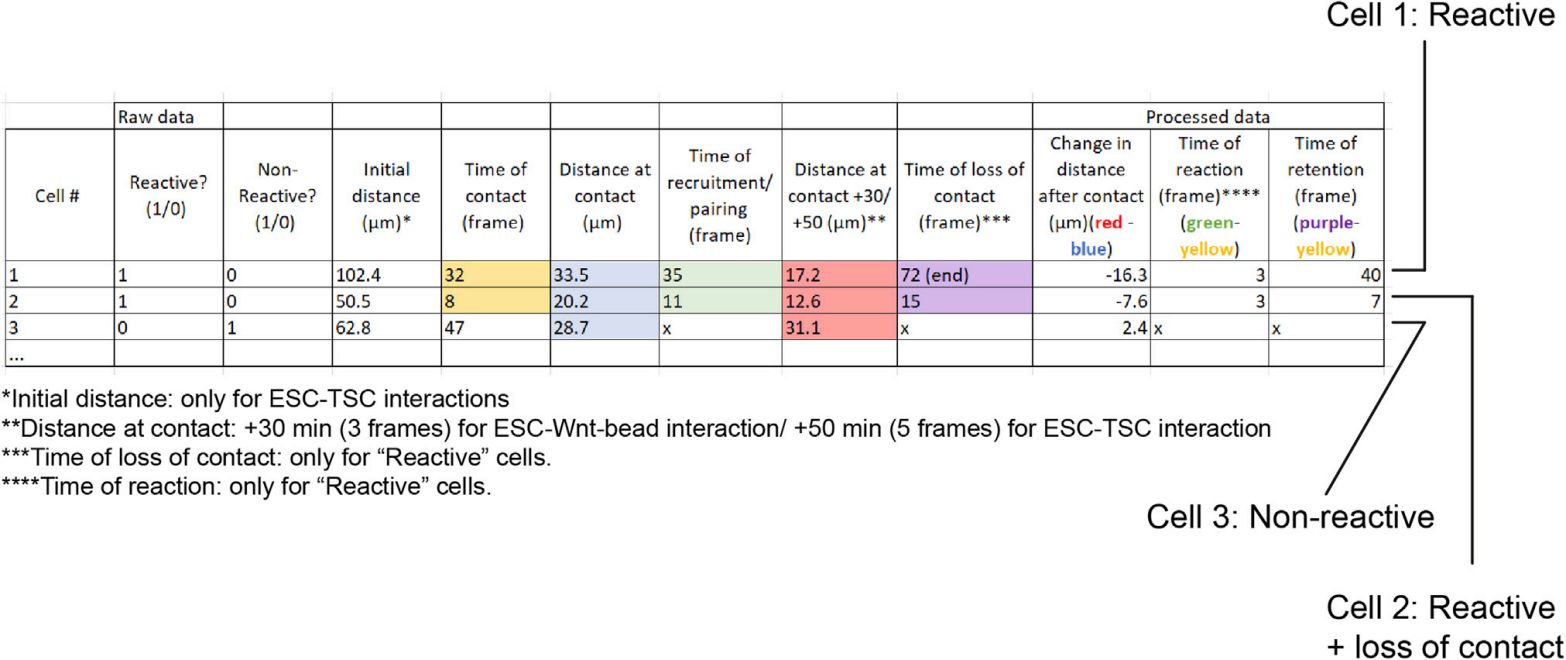
Example of reactivity quantification Columns indicate specific measurements performed in the protocol and in Figures 4 and 5.

All image quantification is performed in Fiji (ImageJ), and the step-by-step pipeline of analysis is described below (exemplified in Figures 4 and 5). Raw data processing is performed in Excel, and an example of the layout is shown below (Figure 6). Once processed, data representation and statistical analysis is performed in Prism version 8 or later (GraphPad).

10. Identifying cells of interest and initial quantification:
  a. Using the Ftractin-mRuby3 signal, identify ESCs or primed ESCs that interact with a bead or TSC via a cytoneme.

***Optional:*** Crop these cells of interest from the larger field of view for ease of analysis and minimizing storage requirements.

***Note:*** Exclusion criteria: apoptotic/non-adherent cells, non-cytoneme-mediated interactions resulting from cell or bead movement, and interactions through cytonemes that are already in contact with a bead or TSC at the start of video are excluded from the analysis at this stage.

11. Quantification of the interactions between ESC/primed ESC cytonemes and sources of Wnt (Wnt-beads or TSCs) (Figures 4 and 5, specific measurements are indicated in the figures).
  a. Observe the interaction between the ESC/primed ESC and the source of Wnt. Aim to identify the first contact between the cell and the source of Wnt, usually mediated by thin cytonemes (most clearly seen using the mRuby3 signal).
  b. For all measurements listed below, record the values in an Excel sheet, one row per interaction analyzed (Figure 6, columns indicate specific measurements listed below).
  c. Only for ESC/primed ESC-TSC interactions: Using the “Line” tool in Fiji, measure and register the initial distance between the ESC/primed ESC and the TSC (“Initial Distance”).
  d. Record the frame in which the initial interaction occurs (“Time of contact”). Additionally, use the “Line” tool and measure the distance between the center of the ESC/primed ESC and the source of Wnt (the Wnt bead or the center of the TSC) at the time of interaction (“Distance at contact”).
  e. Framewise, determine if contact is maintained, resulting in either the bead being recruited towards the soma of the ESC/primed ESC, or in ESC/primed ESC-TSC pairing. This is categorized as a “Reactive interaction”. Record the frame where recruitment/pairing occurs (“Time of recruitment”).
  f. If contact is not maintained, or if no subsequent recruitment/pairing occurs, the interaction is categorized as “Non-reactive interaction”.
  g. For both “Reactive” and “Non-reactive” interactions, use the “Line” tool to measure and register the distance between the ESC/primed ESC and the source of Wnt in frames after contact: For ESC/primed ESC-Wnt-bead interaction measure the distance 3 frames (30 min) after contact (“Distance at contact+30”); for ESC/primed ESC-TSC pairing, measure the distance 5 frames (50 min) after contact (“Distance at contact+50”).
    ***Note:*** We find that 30 min (for ESC/primed ESC-Wnt-bead interaction) and 50 min (for ESC/primed ESC-TSC pairing) are enough to reliably capture the approximation between the ESC/primed ESC and the source of Wnt following initial contact (in “Reactive interactions”), or lack thereof (in “Non-Reactive Interactions”).
  h. Continue monitoring the interaction between the ESC/primed ESC and the source of Wnt. If ESC/primed ESC-Wnt source contact is lost, register the time of loss of contact (“Time of loss of contact”). If contact is maintained until the end of the video, register that time.
  i. Analyze each interaction following these criteria, aiming to analyze at least 30 cells from each experiment (i.e., each video).

12. Raw data processing, post-analysis and data representation (Figure 6).
  a. Pool cells from ≥ 3 independent experiments for post-analysis.
  b. Calculate the percentage of “Reactive Interactions” and the percentage of “Non-Reactive Interactions” of the total observed interactions.
  c. Calculate the “Change in distance after contact” by subtracting the “Distance at contact” from the “Distance at contact+30” (ESC/primed ESC-Wnt-bead interaction) or “Distance at contact+50” (ESC/primed ESC-TSC pairing).
  d. For “Reactive interactions” only, calculate the “Time of Reaction” (time required for a cell to pair with or uptake the source of Wnt after initial contact) by subtracting the “Time of contact” from the “Time of pairing”.
  e. Calculate the “Time of Retention” by subtracting the “Time of contact” from the “Time of loss of contact”.
  f. For all frame-based measurements, convert the values to minutes (1 frame = 10 min).
  g. Repeat analysis and post-analysis for all conditions examined (e.g., ESCs or primed ESCs interacting with control TSCs vs ESCs or primed ESCs interacting with IWP2-treated TSCs) and use Prism (GraphPad) for data presentation and comparison between conditions by statistical significance (described under “quantification and statistical analysis”).

### Imaging Wnt-mediated Ca^2+^ transients in the cytonemes of ESCs or primed ESCs

**Timing: 10–12 h**

This protocol describes how to set up and image a slide of GCaMP6s-expressing ESCs or primed ESCs contacting Wnt3a-beads with cytonemes. For this, GCaMP6s-expressing ESCs or primed ESCs are pre-seeded in imaging slides for 4–6 h and transferred to a microscope. Wnt3a-beads are added whilst cells are *in situ,* to capture the initial stages of Wnt3a-bead interaction with the cytonemes. Imaging is performed every 5 s for 20 min for the same cell. The resulting images are used to measure the generation of Wnt-mediated localized Ca^2+^ transients in the cytonemes of ESCs. Variations of this protocol include adding Kainate (100 μM), CNQX (10 μM) or other drug treatments to the media (added after step 14, below), or the use of control-beads (e.g., iWnt3a-beads).

13. Prepare a suspension of GCaMP6s-expressing ESCs or primed ESCs.
  a. Starting from a 6-well plate culture of ESCs or primed ESCs stably expressing GCaMP6s (prepared as described under “before you begin”), aspirate culture media and rinse once with PBS.
  b. Add 0.5 mL 0.25% Trypsin-EDTA and return the plate to the incubator.
  c. Incubate for ∼4 min at 37°C, 5% CO_2_ until cells are detached.
    **CRITICAL:** Do not incubate cells with 0.25% Trypsin-EDTA for longer than 5 minutes to minimize possible receptor cleavage.
  d. With a 5 mL serological pipette, use 2 mL ESC basal media to resuspend the cells. Transfer cell suspension to a 15 mL Falcon tube.
  e. Pellet cells by centrifugation at 1.2 **×** 10^3^ rcf for 4 min at RT.
  f. Aspirate supernatant, carefully avoiding the pelleted cells.
  g. Resuspend pelleted cells in 1 mL ESC basal media by repeatedly pipetting up and down.
  h. Using a glass hemocytometer, estimate the concentration of cell in the suspension.

14. Seed GCaMP6s-expressing ESCs or primed ESCs for imaging.
  a. Dilute ESCs or primed ESCs to 7,000 cells per 250 μL in ESC basal media.
  b. Transfer cell mixture to a well of an ibiTreat μ-Slide 8 well slide (IBDI, cat. Num. 80826). Prepare several wells.
  c. Return to incubator and incubate for 4–6 h at 37°C, 5% CO_2_ until cells are attached and generate cytonemes.
15. Initial setup to image GCaMP6s-expressing ESCs or primed ESCs contacting Wnt3a-beads.
  ***Optional:*** Transfer cells to a pre-warmed polystyrene box for transport to the spinning disk microscope.
  a. Pre-stabilize the incubation box of the spinning disk microscope at 37°C, 5% CO_2_
  b. Place the cell-containing slide in the motorized stage.
  c. Select the 20**×**/0.75 objective (or equivalent).
  d. Perform Kohler illumination adjustment of the transmitted light following standard procedures.

16. Add Wnt3a-beads (or control-beads, e.g., inactivated Wnt3a-beads, Wnt5a-beads or BSA-beads) to the slide. See Preparation of Wnt3a-beads and control beads and Wnt/β-catenin pathway activation assay and (Habib et al., 2013; Lowndes et al., 2017)
  a. Prepare a tube with 25 μL ESC basal media.
  b. Transfer 1 μg Wnt3a-beads (or control-beads, such as inactivated Wnt3a-beads, Wnt5a-beads or BSA-beads) to the tube with media and mix.
    **CRITICAL:** to ensure homogenization and single-bead suspension, triturate stock Wnt3a-bead solution by pipetting up and down for >100 times using a ∼100 μL volume. This point is critical, as if skipped, beads will clump together in big clusters.
  c. Add the diluted Wnt3a-bead solution to the well of GCaMP6s-expressing ESCs or primed ESCs, dropwise.
  d. Incubate for ∼15 min to allow the Wnt3a-beads to reach the bottom of the well.

17. Image GCaMP6s-expressing ESCs or primed ESCs contacting Wnt3a-beads
  a. Using brightfield, chose a cell that is contacting a Wnt3a-bead with a cytoneme. Use the GCaMP6s channel to visualize the ESCs or primed ESCs and find an optimal z-plane that allows stable imaging of the interaction. Only one position containing one or a few cells is imaged at a time.
  b. Adjust laser intensities and exposure times to be as low as possible to avoid phototoxicity. Troubleshooting 4.
  c. Set up the time-lapse imaging protocol: one image every 5 s, for 20 min.
    ***Optional:*** Image a one-cycle loop to check that the setup is correct.
  d. Run imaging pipeline, capturing images for brightfield and GCaMP6s (GFP channel) at every loop.
  e. Once finished, choose another cell/position and repeat imaging.
  f. After 4–5 cells/positions (approx. 1 h), change to a new well and repeat steps 16 and 17. This way the initial interaction between Wnt3a-beads and ESCs or primed ESCs will be observed.
  g. Perform analysis in the images obtained (see Quantifying localized Ca2+ transients on ESC or primed ESC cytonemes during interaction with a Wnt source).

### Quantifying localized Ca^2+^ transients on ESC or primed ESC cytonemes during interaction with a Wnt source

**Timing: 3–6 h**

**Figure 7:**
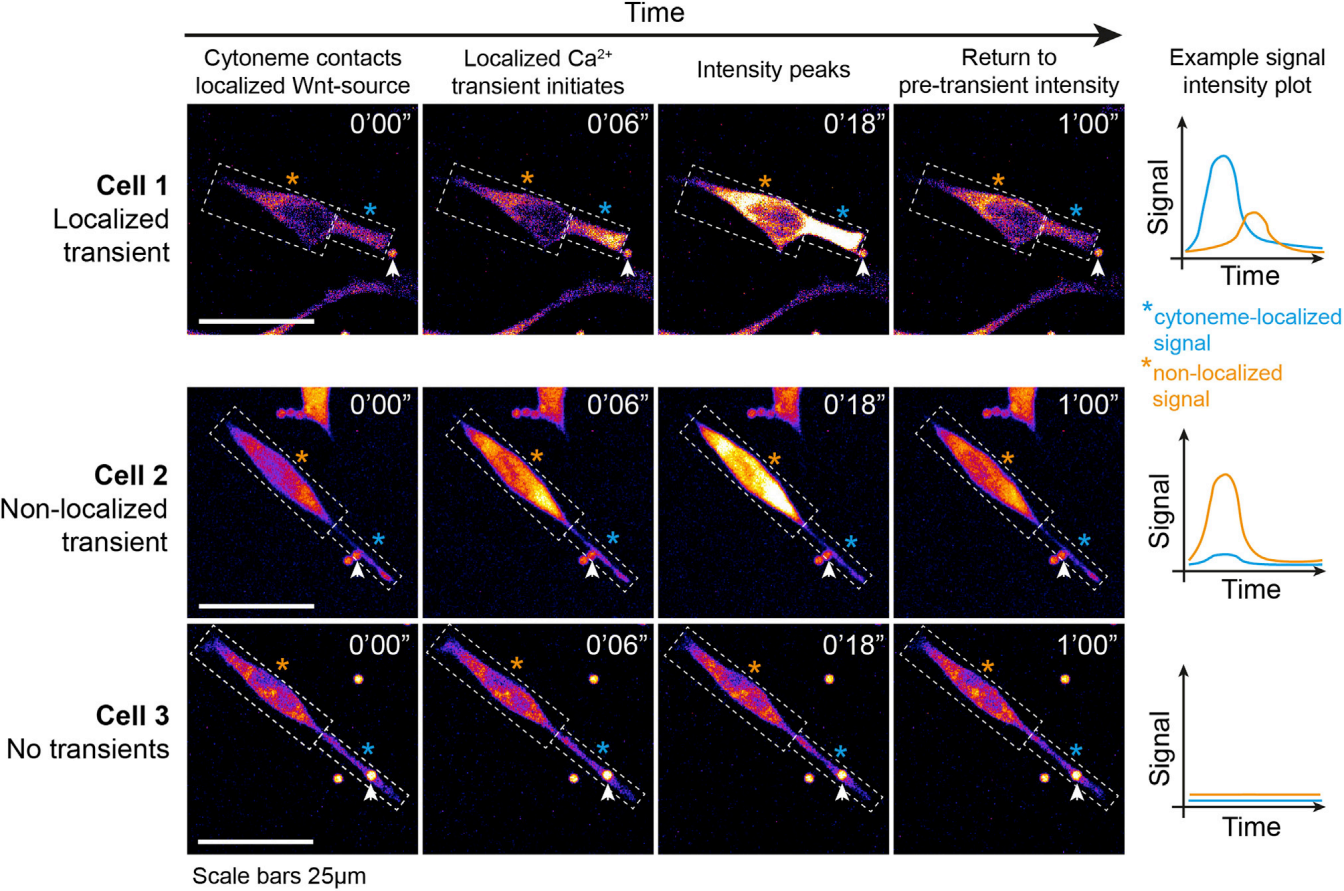
Representative frames of a time-course imaging showing an ESC contacting a Wnt3a-bead (white arrowhead) with a cytoneme (dashed box & blue asterisk) Dashed box & orange asterisk designates the non-localized portion of the cell. GCaMP6s intensity is displayed using the “Fire” LUT (Fiji, ImageJ). Examples are shown for a cell that generates a localized Ca^2+^ transient in the cytoneme (Cell 1, *top*), a cell that generates a non-localized Ca^2+^ transient (Cell 2, *middle*) and a cell without a Ca^2+^ transient (Cell 3, *bottom*). Example signal intensity plots for the localized (dashed box, blue asterisk and plot) and non-localized (dashed box, orange asterisk and plot). Time is displayed in seconds and minutes. Scale bars, 25 μm.

All image quantification is performed in Fiji (ImageJ), and the step-by-step pipeline of analysis is described below. Raw data processing is performed in Excel, and an example of the layout is shown below (Figure 7). Once processed, data representation and statistical analysis is performed in Prism (GraphPad).

18. Identifying cells of interest
  a. Use the GCaMP6s signal to identify single ESCs or primed ESCs that contact Wnt-bead with a cytoneme. Following Imaging Wnt-mediated Ca2+transients in the cytonemes of ESCs or primed ESCs), at least one cell per position should be contacting a Wnt3a-bead.
    ***Optional:*** Crop these cells of interest from the larger field of view for ease of analysis and minimizing storage requirements.
  b. Interactions resulting from cell motility or bead movement (i.e., non-cytoneme mediated interactions) are excluded from the analysis.

***Note:*** apoptotic/non-adherent cells are also excluded from the analysis.

**Figure 8:**
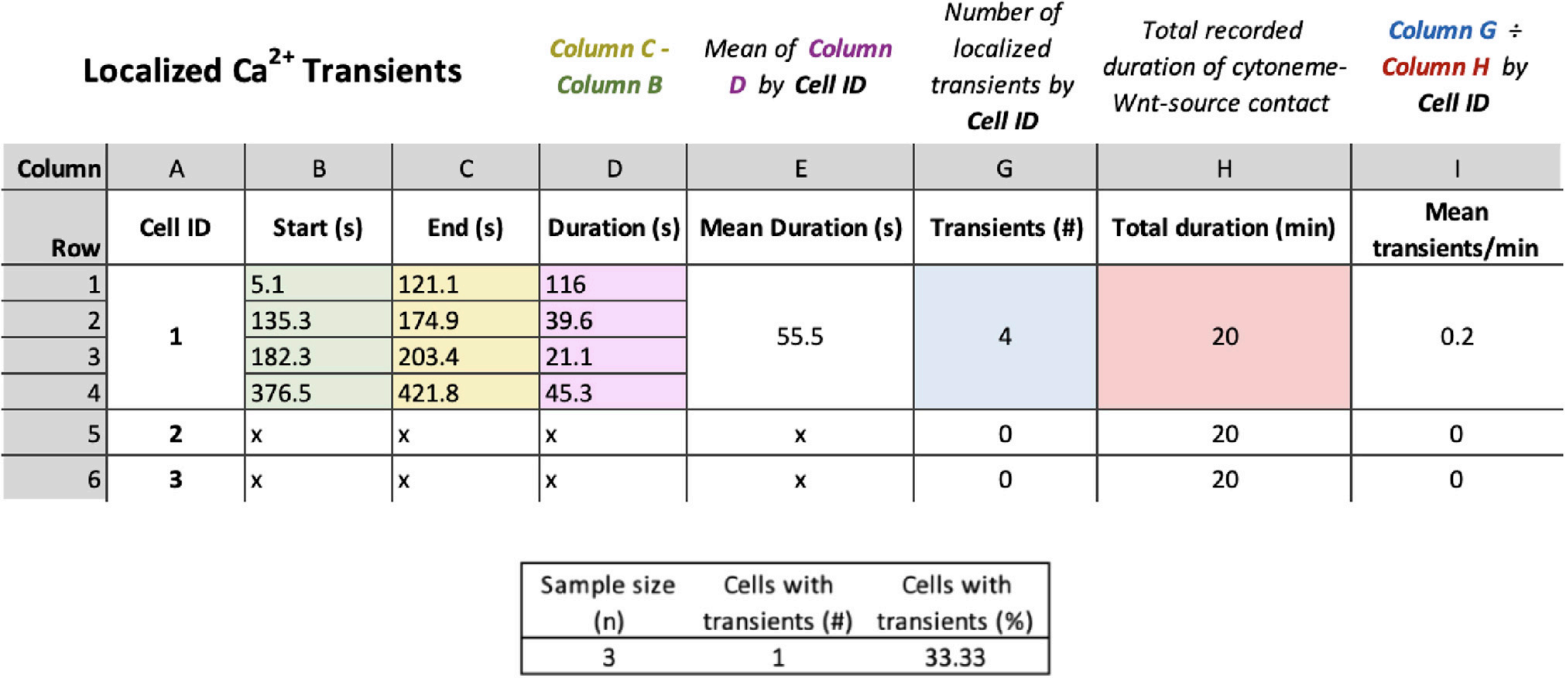
Example of localized Ca^2+^ transient quantification Columns indicate specific measurements performed in the protocol.
  f. With the total recorded time of cytoneme-Wnt-source contact (“Total duration”), the mean number of transients per cell per minute (“Mean transients/min”) can be calculated.
  g. From the total number of cells included in the analysis (“Sample size (n)”), and the number of cells with >0 transients (“Cells with transients (#)”), the “Cells with transients (%)” can then be calculated (Figure 8).

19. Quantifying localized Ca^2+^ transients on ESC/primed ESC cytonemes
  a. View the GCaMP6s signal using a high-contrast LUT, such as ‘Fire’ in ImageJ.
  b. Ca^2+^ transients are identified qualitatively as a sharp increase in GCaMP6s signal, typically over the course of <30 s, which then diminishes to the basal level.
  c. Cytoneme-localized transients (referred to as ‘localized transients’) are manually distinguished from whole-cell transients or those emanating from the cell-soma (see expected outcomes). Localized transients arise from the contact area between cytoneme and Wnt-source, and may emanate to the rest of the cell, or remain in the cytoneme (Figure 7).
  d. For each localized transient, record the start (“Start”, GCaMP6s signal increases) and end (“End”, GCaMP6s signal returns to basal level) time points.
  e. From this data, the duration of each localized transient (“Duration”), the mean duration of all transients occurring within each cell (“Mean Duration”) and the number of localized transients per cell (“Transients (#)”) can be calculated (see Figure 8 for an example of data organization).

## Expected outcomes

This protocol describes how to analyze the reactivity of the ESC or primed ESC cytonemes to localized Wnt by either measuring the interaction between ESC cytonemes and Wnt sources or measuring the generation of localized Ca^2+^ transients in response to Wnt contact.

In the first part of the protocol, the step-by-step procedure enables analysis of ESC/primed ESC cytoneme reactivity to localized sources of Wnt (Wnt3a-beads or TSCs). The experiments described will result in four dimensional images of ESCs generating cytonemes and interacting with Wnt3a-beads or TSCs. When analyzed according to the “quantifying cytoneme reactivity to sources of Wnt (Wnt3a-beads or TSCs)” section, ESC/primed ESC reactivity to the localized sources of Wnt can be quantified. In the majority of ESCs, initial contact of a Wnt-source by a cytoneme will be followed by Wnt recruitment (Wnt3a-beads) or cell pairing (TSCs). In these cells the “Change in distance after contact” will be negative, indicating a reduction in the distance between the cells following contact. However, when presented with inactivated Wnt3a-beads or with TSCs with inhibited Wnt secretion (IWP2-treated TSCs), most ESC cytoneme-mediated contacts should not be followed by recruitment or pairing. As a result, the “Change in distance after contact” in these conditions should remain, in average, around 0. On the other hand, primed ESCs non-selectively recruit both Wnt3a-beads and control-beads following initial cytoneme contact but fail to pair with TSCs (both control and IWP2 treated) upon initial cytoneme-mediated interaction.

When performing this protocol some common pitfalls should be avoided. Care should be taken to ensure maximal cell viability. For instance, the duration of trypsin incubation should be reduced to a minimum and cells should be kept in stable incubation conditions (37°C, 5% CO_2_) whenever possible. If cell manipulation is suboptimal, dead or dying cells will be apparent in the imaging. Optimal cell concentration during imaging is also important. The number of ESCs/primed ESCs in the well should be high enough to facilitate contact with the sources of Wnt (Wnt3a-beads or TSCs), but low enough to avoid cell clumping and excessive ESC-ESC or primed ESC-primed ESC contact. Finally, careful and consistent analysis should be performed. Standardized decisions on what is considered a “Reactive” and a “Non-reactive” interaction should be established from preliminary experiments. We recommend following the criteria described in the “quantifying cytoneme reactivity to sources of Wnt (Wnt3a-beads or TSCs)” section, and the representative images shown in Figures 4 and 5 or previous publications (Junyent et al., 2020, 2021a). Decision making can be supported by measuring the change in distance between ESCs/primed ESCs and the source of Wnt, as described.

In the second part of the protocol, we describe the steps to prepare and image a slide of GCaMP6s-expressing ESCs or primed ESCs, and the generation of Wnt-mediated Ca^2+^ transients in their cytonemes. When performed correctly, ESCs or primed ESCs will make contact with Wnt3a-beads with outstretched cytonemes, as with the Reactivity analysis. In ESCs, localized Ca^2+^ transients - ‘flashes’ of higher intensity GCaMP6s signal - should be observed initiating in the bead-contacting cytonemes. Localized Ca^2+^ transient generation is reduced in primed ESCs contacting Wnt3a-beads, or in ESCs treated with CNQX (10 μM), lacking the Wnt/β-catenin pathway co-receptors Lrp5/6 or contacting iWnt3a-beads. Critical for this protocol is the careful and standardized distinction of localized from non-localized Ca^2+^ transients. Ca^2+^ transients that initiate in the cytonemes, particularly those near the contact point with the Wnt-source, are categorized as localized. While these may also emanate to the rest of the cell, they are distinguished from non-localized transients that do not arise from the Wnt-contacting cytoneme. Non-localized transients frequently occur across the entire cell simultaneously or originate from the cell soma (see Figure 7). Care should be taken to distinguish increases in signal intensity in bead-contacting cytonemes that are a result of a cell-wide transient (i.e., a non-localized transient – Figure 7, Cell 2) from similar increases in signal intensity that results from the bead-contacting cytoneme (i.e., localized transients – Figure 7, Cell 1) prior to any subsequent cell-wide transients. We recommend following the steps described in “quantifying localized Ca2+ transients on ESC or primed ESC cytonemes during interaction with a Wnt source”.

Together, this protocol enables the characterization of key functions of the ESC cytonemes, and their comparison with the cytonemes formed by primed ESCs. When combined with the use of KO ESC lines or drug treatments, this protocol will provide novel insights into the mechanisms stem cells use to contact and interact with localized sources of Wnt.

In addition to the protocols described here, we have performed further experiments to explore the composition and functionality of the ESC cytonemes. These include immunostaining of the cytonemes with antibodies against key molecular factors, such as cytoskeletal proteins (αTubulin, F-actin), Wnt co-receptors and Wnt/β-catenin pathway components (Lrp6, β-catenin) and ionotropic glutamatergic receptors. For these, cells are co-cultured with Wnt3a-beads as described in the protocol and fixed with paraformaldehyde. Then, standard immunofluorescence protocols are followed. Also, we have previously shown that Wnt transport through cytoneme-mediated interactions between ESCs and TSCs is an important first step to ensure correct synthetic embryogenesis. To complement the protocols described here, ESC-TSC synthetic (ETS) embryos could also be prepared, following other published protocols (Harrison et al., 2018). Briefly, to generate ETS embryos, ESCs or primed ESCs would be co-cultured with TSCs in a Matrigel culture platform, with structure formation analyzed at specific timepoints. In the accompanying publication, we saw that inhibition of Wnt production by IWP2 treatment or CNQX treatment reduces the capacity of ESCs to form synthetic embryos. Formation of ETS embryos with primed ESCs is also reduced. Although detailed elsewhere, these experiments can also prove valuable to explore cytoneme function.

## Quantification and statistical analysis

All image quantification is performed in Fiji (ImageJ), and the step-by-step pipeline of analysis is described in the protocol. Raw data processing is performed in Excel, and examples of the layout are shown in Figures 6 and 8. Once processed, data representation and statistical analysis is performed in Prism version 8 or later (GraphPad). Statistical comparisons should be performed with adequate statistical tests (e.g., Fisher’s exact test for qualitative data such as Percentage of “Reactive Interactions”, and T-test for continuous data such as “Change in distance after contact”).

## Limitations

When investigating the cell-cell interactions in the formation of synthetic embryos, the multiplicity of a spectrum of signals received by the ESC cytoneme cannot be ruled out. This factor can be overcome through the use of the Wnt3a-beads as a recapitulation of localized Wnt3a ligands alone. Further, cellular populations of both ESCs and TSCs are heterogenous (Frias-Aldeguer et al., 2019; Ying et al., 2008), hence sufficient sampling of the cellular population is required. Sample-size and statistical power calculations can be performed following preliminary experiments. Expected mean and deviation values will be required. These calculations will dictate the minimal sample required. Alternatively, post-hoc power calculations can be performed to ensure that reliable statistical differences have been achieved. Of note, we analyze ≥ 30 interactions per condition, pooled at relatively equal proportions from ≥ 3 independent experiments (different cells, different imaging sessions).

Recently, several papers have been published that demonstrate the culture of ESCs populations between naïve and primed (Kinoshita et al., 2020; Neagu et al., 2020). Our protocols were established before their publication and therefore do not discuss the use of these ‘rosette’ or ‘formative’ ESC states. However, analysis of Wnt-reactivity and Ca^2+^ transients may easily be adapted for the study of these pluripotency states and other cell types. In terms of cell culture conditions for ESCs and TSCs, while this protocol includes serum, it may be adapted to serum free media. Importantly, genetic KOs may affect pluripotency of ESCs, hence it is important to maintain such ESCs in 2i culture prior to synthetic embryogenesis or cytoneme reactivity analysis. The efficiency of imaging may be improved by cell-cycle synchronization of cells, however care should be taken and controls should be included to rule out interference of the synchronizing agent with cytoskeletal organization and cytoneme function. The ESC-TSC interaction protocol we have used in our experiments (Junyent et al., 2020) (Junyent et al., 2021a) takes place in 2D culture. Development of 3D culture methods may better recapitulate the *in vivo* environment at the expense of increasing biological, imaging, and analytical complexity.

Sufficient Wnt3a-bead activity is crucial for these measurements. Therefore, validating the purity and the activity of soluble Wnt3a and subsequently the Wnt3a-bead activity using the LSL assay are critical steps. Variation in the activity of individual Wnt3a-beads activity may exist within batches, however the activity of individual beads cannot be measured. This limitation is overcome by the sufficient sampling of multiple ESC-bead interactions to be representative of the population.

In terms of the confocal imaging and subsequent analysis, as with many fluorescent imaging techniques, photobleaching and phototoxicity can arise as live-cell imaging takes place, particularly over longer durations. This can be minimized using the lowest possible exposure time and illumination power to capture sufficient signal. Quantification of Ca^2+^ transients requires that interactions of interest are manually screened to distinguish whole-cell transients (non-localized) from those localized to the region of bead-cytoneme interaction. Hence, these measurements are taken at the single-cell level, and currently precludes an automated, populational quantification.

## Troubleshooting

### Problem 1

Cell density is not optimal for analysis (for an example see Figures 2 and 3). Related to “Measuring cytoneme-mediated communication between ESCs or primed ESCs and TSCs” step 3., or “Measuring cytoneme-mediated recruitment of Wnt-beads by ESCs or primed ESCs” step 7.

### Potential solution

Optimize cell seeding density. Aim to get single ESCs/primed ESCs close to one or two small TSC clusters, or single ESCs/primed ESCs with a few (3–5) Wnt3a-beads within reach (∼10–25 μm) of a cytoneme. This can be achieved by increasing or decreasing the concentration of ESCs/primed ESCs, TSCs or Wnt3a-beads.

### Problem 2

Presence of dead cells or debris during imaging. Related to “before you begin” steps 3. and 9., “Measuring cytoneme-mediated communication between ESCs or primed ESCs and TSCs” steps 1, 2. or “Measuring cytoneme-mediated recruitment of Wnt-beads by ESCs or primed ESCs” step 6.

### Potential solution

Increased care should be taken to ensure cells are incubated with Trypsin-EDTA for a short period of time (<4 min), and that cell-containing plates or slides are kept at 37°C, 5% CO_2_ whenever possible. Unstable incubation systems (e.g., temperature lower than 37°C, CO_2_ concentrations too low) will affect viability. Transport times to the microscope can also affect cell viability. We recommend using a pre-warmed polystyrene box for transport.

### Problem 3

Low GCaMP6s or Ftractin-mRuby3 intensity or presence of construct-negative cells in the imaging. Related to “Preparation of ESC lines expressing the Ca2+ sensor GCaMP6s or the F-actin reporter Ftractin-mRuby3" step 14.

### Potential solution

To ensure homogeneous expression of fluorescent constructs (e.g., GCaMP6s, Ftractin-mRuby3), FACS can be used. A population of ESCs stably-expressing high levels of the construct can be sorted and expanded. If low-expressing cells or negative cells are detected during imaging, a second round of sorting can be performed.

### Problem 4

High temporal-resolution imaging required for Ca^2+^ transient imaging induces photo/fluorotoxicity. Related to “Imaging Wnt-mediated Ca2+ transients in the cytonemes of ESCs or primed ESCs” step 17.

### Potential solution

The need for high exposure times or fluorescent illumination intensity can be limited during high-temporal resolution microscopy by ensuring that the expression of GCaMP6s and Ftractin-mRuby3 is sufficiently high that low exposure times and illumination power can be used (see problem 3). Imaging cells of interest in series, with a confocal microscopy (i.e., non-epifluorescence illumination), ensures that cells are not exposed to high intensity light until necessary.

### Problem 5

The activity of Wnt3a beads is low. Related to “before you begin” step 18.

### Potential solution

See Lowndes et al. 2017 for a comprehensive trouble shooting of this problem.

## Resource availability

### Lead contact

Further information and requests for resources and reagents should be directed to and will be fulfilled by the lead contact, Dr. Shukry J. Habib (shukry.habib@kcl.ac.uk).

### Materials availability

This study did not generate new unique reagents but used previously published reagents. To access them, directly contact the source lab.

## Data Availability

This study did not generate/analyze new data sets.
